# The Influence of Higher-Order Epistasis on Biological Fitness Landscape Topography

**DOI:** 10.1007/s10955-018-1975-3

**Published:** 2018-02-07

**Authors:** Daniel M. Weinreich, Yinghong Lan, Jacob Jaffe, Robert B. Heckendorn

**Affiliations:** 1 Department of Ecology and Evolutionary Biology, Brown University, Providence, RI 02912 USA; 2 Center for Computational Molecular Biology, Brown University, Providence, RI 02912 USA; 3 Computer Science Department, University of Idaho, 875 Perimeter Drive, MS 1010, Moscow, ID 83844 USA

**Keywords:** Higher-order epistasis, Fitness landscapes topography, Natural selection, NK landscape, Sequence space combinatorics

## Abstract

The effect of a mutation on the organism often depends on what other mutations are already present in its genome. Geneticists refer to such mutational interactions as epistasis. Pairwise epistatic effects have been recognized for over a century, and their evolutionary implications have received theoretical attention for nearly as long. However, pairwise epistatic interactions themselves can vary with genomic background. This is called higher-order epistasis, and its consequences for evolution are much less well understood. Here, we assess the influence that higher-order epistasis has on the topography of 16 published, biological fitness landscapes. We find that on average, their effects on fitness landscape declines with order, and suggest that notable exceptions to this trend may deserve experimental scrutiny. We conclude by highlighting opportunities for further theoretical and experimental work dissecting the influence that epistasis of all orders has on fitness landscape topography and on the efficiency of evolution by natural selection.

**Electronic supplementary material:**

The online version of this article (10.1007/s10955-018-1975-3) contains supplementary material, which is available to authorized users.

## Introduction

One of the more evocative pictures of biological evolution is that of a population climbing the fitness landscape [37, 44]. This image was originally proposed by Wright [73] to build intuition into his [72] and Fisher’s [19] technical treatment of Darwin’s theory of natural selection in finite populations under Mendelian genetics [51]. The topography of the fitness landscape represents the strength and direction of natural selection as local gradients that influence the direction and speed with which populations evolve.

While several distinct framings of the fitness landscape have been suggested [51], here we employ the projection of genotypic fitness over Maynard Smith’s sequence space [36]. Sequence space is a discrete, high-dimensional space in which genotypes differing by exactly one point mutation are spatially adjacent. Thus, proximity on the fitness landscape corresponds to mutational accessibility, and selection will try to drive populations along the locally steepest mutational trajectory. (See [68] for several processes not readily captured by this construction.)

The most obviously interesting topographic feature of the fitness landscape is the number of maxima, a point already recognized by Wright [73]. Two (or more) maxima can constrain natural selection’s ability to discover highest-fitness solutions, since populations may be required to transit lower-fitness valleys on the landscape en route. (Though see [25, 65] for the population genetics of that process, sometimes called stochastic tunneling [15, 25].)

### Epistasis and Fitness Landscape Topography

Epistasis is the geneticist’s term for interactions among mutational effects on the organism [46]. For example, genetically disabling two genes whose products act in the same linear biochemical pathway can have a much more modest effect than the sum of the effects of disabling either gene in isolation. Alternatively, disabling two functionally redundant genes can have a much more substantial effect than expected. (Indeed, such observations have taught us quite a bit about the organization of biochemical pathways, e.g., [2].)

Epistatic interactions between mutations can occur for any organismal trait, including fitness. Importantly, epistasis for fitness has an intimate connection to the topography of the fitness landscape, a fact also already appreciated by Wright [73]. For example, multiple peaks require the presence of mutations that are only conditionally beneficial (called sign epistasis [49, 68]). More generally, an isomorphism exists between fitness landscapes defined by mutations at some *L* positions in the genome and the suite of epistatic interactions possible among them. This follows because, while any particular mutation can appear on 2$^{L-1}$ different genetic backgrounds (assuming two alternative genetic states, or alleles, at each position), each such mutation-by-background pair corresponds to a distinct adjacency in sequence space. Consequently, arbitrary differences in the fitness effect of a mutation across genetic backgrounds can generically be represented on the fitness landscape [68].

### Higher Order Epistasis and Fitness Landscape Topography

Widespread epistasis between pairs of mutations has been recognized in nature for over 100 years [46, 67], and the corresponding evolutionary theory is fairly advanced (e.g., [5, 71]). However, pairwise interactions can themselves vary with genetic background, called higher-order epistasis [13, 67]. And while it is now becoming clear that higher-order interactions are commonplace in nature [32, 42, 61, 67], their influence on natural selection is less well understood (though see [55]). Here, we present a simple framework for assessing the influence on fitness landscape topography of epistatic terms of arbitrary order. We speculate that epistatic influence on the topography of naturally occurring fitness landscapes should decline with epistatic order. We tested this prediction using 16 published biological fitness landscapes.

## Methods

### The Order of Epistatic Interactions

Any set of *L* biallelic loci defines $2^{L}$ genotypes, each with $2^{L}$ potentially independent fitness values. Simultaneously, there are $$\left( {{\begin{array}{l} L \\ k \\ \end{array} }} \right) $$ distinct subsets of *k* mutations that in principle can also independently contribute to a genotype’s fitness. In total, there are thus $$\sum \nolimits _{k=0}^L \left( {{\begin{array}{l} L \\ k \\ \end{array} }} \right) $$ = 2$^{L}$ subsets of mutations (i.e., the power set of *L* mutations). This counting reflects the isomorphism between any fitness landscape and its corresponding suite of epistatic terms [67].

We designate interactions among any subset of *k* mutations as $k{\mathrm{th}}$-order epistasis. Note that here first-order “epistasis” is degenerate in the sense that it represents the fitness effects of each of the *L* mutations in isolation. And our zeroth-order “epistatic” term is the benchmark, relative to which the effect of each subset of mutations is computed.

**Fig. 1:**
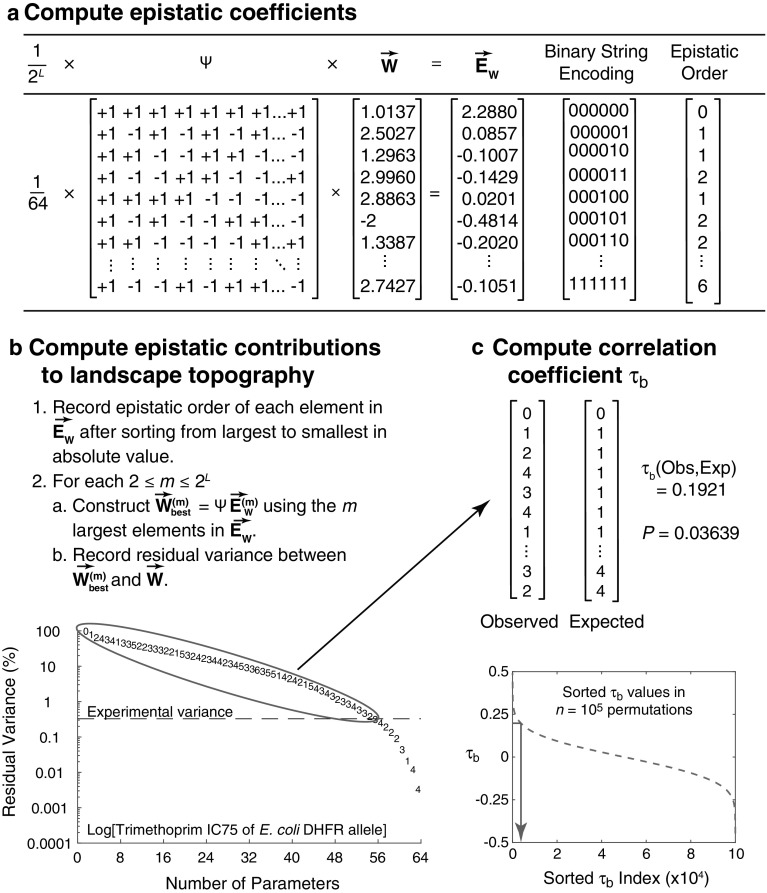
Analytic pipeline, illustrated with data from Palmer et al. [45]. **a** For each dataset, published fitness data (or a suitable proxy, written $\overrightarrow{W}$) were first converted to the corresponding epistatic terms ($\vec {E}$ using the Fourier–Walsh transformation (Eq. 1). **b** Explanatory power of a succession of models using only the *m* largest epistatic terms in absolute value ($\overrightarrow{W_{\mathrm{best}}^{\left( m \right) }}$) were compared with the published data. For given value of *m*, these models provably have the greatest explanatory power (smallest residual variance) of any model with exactly *m* parameters (Appendix). The symbols plotted represent the epistatic order (Sect. 1.2) of each successive parameter added to the model. **c** Rank correlation coefficient $(\tau _{\mathrm{b}})$ between the empirical sequence of epistatic orders and those of our naïve expectation (Eq. 2) were computed. In cases where experimental variance was reported, these sequences were truncated as soon as the remaining model variance was less than the experimental variance. For the data shown, that truncation occurred after the $55{\mathrm{th}}$ epistatic term. Finally, statistical significance was assessed by a permutation test that asked whether the observed sequence of epistatic orders was significantly different than random. For the data shown (red arrow), the observed value of $\tau _{\mathrm{b}}$ (0.1921) was smaller than only the 3639 largest of $10^{5}$ values obtained by the permutation test, yielding $P = 0.03639$

### The Fourier–Walsh Transformation

Following earlier work [22, 41, 59, 64, 67] we employ the Fourier–Walsh transformation (Fig. 1a) to convert between fitness landscapes and their corresponding epistatic terms. This is a linear transformation written

1 $$\begin{aligned} \overrightarrow{E_{W}}=\frac{1}{2^{L}}{\varvec{\Psi }}\overrightarrow{W}. \end{aligned}$$

Here $\overrightarrow{W}$ is the vector of all $2^{L}$ fitness values arranged in the canonical order defined by ascending *L*-bit binary numbers encoding the corresponding genotype with respect to the presence or absence of each mutation (e.g., [33]). (*W* is the traditional population genetics symbol for fitness.) ${\varvec{\Psi }}$ is the Hadamard matrix, the unique, symmetric $2^{L} \times 2^{L}$ matrix whose entries are either +1 or −1 and whose rows (and columns) are mutually orthogonal. (${\varvec{\Psi }}$ can be written for arbitrary *L*, as for example with the hadamard() function in the software package Matlab, Mathworks, Natick, MA.) Finally, $\overrightarrow{E_{W}}$ is the resulting vector of 2$^{L}$ epistatic terms arranged in the canonical order defined by ascending *L*-bit binary numbers whose 1’s indicate the corresponding subset of interacting loci. Figure 1a illustrates this transformation using the data in [45]. For example, the fourth component of $\overrightarrow{E_{W}}$ (–0.1429) signals a negative epistatic interaction between the two most 3’ mutations in that dataset. (See Fig. 1 in [54] for a graphical representation of the elements of $\overrightarrow{E_{W}}$, and [50] for the relationship between Eq. (1) and other formalisms for computing epistatic terms.)

The orthogonality and symmetry of ${\varvec{\Psi }}$ means that ${\varvec{\Psi }}^{\mathrm{T}} \cdot {\varvec{\Psi }}={\varvec{\Psi }}^{2}= 2^{L}$**I**, where **I** is the identity matrix. This means that, just as Eq. (1) converts any landscape into its epistatic terms, so too can any vector of epistatic terms $\vec {E}$ be converted into its corresponding fitness landscape as $\overrightarrow{W}={\varvec{\Psi }}\vec {E}.$ We take advantage of this fact next.

### Subsetting Approximations of a Fitness Landscape

Given fitness function $\overrightarrow{W}$, we now introduce subsetting approximations $\overrightarrow{W^{\left( m \right) }}={\varvec{\Psi }}\overrightarrow{E_W^{\left( m \right) } }$. Here, the $\overrightarrow{E_W^{\left( m \right) } }$ are constructed so that 0 $\le m \le$ $2^{L}$ of the components are from $\overrightarrow{E_W }=\frac{1}{2^{L}} {\varvec{\Psi }} \overrightarrow{W}$ (Eq. 1) and the remaining 2$^{L} - m$ components are set to zero. There are thus $2^{2^{L}}$ subsetting approximations for any fitness function $\overrightarrow{W}$ (corresponding to the power set of the 2$^{L}$ epistatic terms in $\overrightarrow{E_{W}})$. As a consequence of the orthogonality of the Fourier–Walsh transformation, the sum of squares distance between fitness function $\overrightarrow{W}$ and subsetting approximation $\overrightarrow{W^{\left( m \right) }}={\varvec{\Psi }}\overrightarrow{E_W^{\left( m \right) } }$ is minimized for given *m* if and only if $\overrightarrow{E_W^{\left( m \right) } }$ uses the *m* largest components in absolute value of $\overrightarrow{E_{W}}$ (see Appendix). We denote these 0 $\le m \le$ 2$^{L}$ best subsetting approximations $\overrightarrow{W_{\mathrm{best}}^{\left( m \right) } }$.

(Subsetting approximations defined by interaction order rather than absolute magnitude of epistatic terms were recently employed elsewhere [55].)

### Quantifying the Influence of Epistatic Terms on Empirical Fitness Landscape Topography

To examine the influence of epistasis on fitness landscape topography as a function of epistatic order, we first used Eq. (1) to compute $\overrightarrow{E_W }$ for each $\overrightarrow{W}$ gleaned from the literature (Sect. 2.7). For each 1 $\le m \le$ 2$^{L}$, we then iteratively constructed each $\overrightarrow{W_{\mathrm{best}}^{\left( m \right) } }$. Finally, for each *m* we recorded the residual variance between $\overrightarrow{W_{\mathrm{best}}^{\left( m \right) } })$ and $\overrightarrow{W}$ (minimized by this subsetting approximation; Sect. 2.3), together with the epistatic order of the $m{\mathrm{th}}$-largest component of $\overrightarrow{E_W }$. Figure 1b illustrates this process.

### Statistics

Our hypothesis is that the influence of an epistatic term on the fitness landscape should decline with epistatic order. Put another way, we expected that after sorting the elements of $\overrightarrow{E_{W}}$ (Eq. 1) by their absolute magnitudes, the associated epistatic orders should be represented by a vector of 2$^{L}$ integers that reads:

2 $$\begin{aligned} 0, 1, 1, {\ldots }, 2, 2, 2, {\ldots }, {\ldots }L -- 1, L -- 1, {\ldots }, L. \end{aligned}$$

Specifically, this vector consists of one zero, followed by *L* ones, $$\left( {{\begin{array}{l} L \\ 2 \\ \end{array} }} \right) $$ twos and in general $$\left( {{\begin{array}{l} L \\ k \\ \end{array} }} \right) \quad k$$’s for all 0 $\le k \le$ 2$^{L}$.

We tested this hypothesis for each dataset by first computing Kendall’s $\tau _{\mathrm{b}}$ correlation coefficient [28] between this expectation and the epistatic orders observed among the elements in $\overrightarrow{E_{W}}$ sorted by absolute magnitude. $\tau _{\mathrm{b}}$ is one (negative one) when the observed epistatic orders are perfectly correlated (anticorrelated) with expectation, and zero when they are uncorrelated. Note that Kendall’s $\tau _{b}$ statistic is appropriate because it accommodates ties. For studies that also reported experimental variance, we computed the correlation coefficient after discarding the epistatic orders of all *j*elements in $\overrightarrow{E_{W}}$ that reduced residual variance by less than experimental variance (see Fig. 1b and Table 1) as well as the last *j* epistatic order values in our expectation (given by Eq. 2).

**Table 1:** Analyses of published combinatorially complete empirical and simulated (*NK*) fitness landscapes, sorted by *P* value associated with Kendall’s $\tau _{\mathrm{b}}$

Phenotype [citation]	Number of loci (*L*)	Number of maxima	Number of epistatic terms significantly different from zero$^{\mathrm{a}}$	Kendall’s $\tau _{b}$ correlation coefficient	*P* value$^{\mathrm{b}}$
Log[*S. cerevisiae* HSP90 mutant growth rates] [4]	6	4	41	0.6202	< 0.00001***
Log[diploid S*. Cerevisiae* mutant growth rate] [21]	6	4	3	0.6667	<0.00001***
Log[*E. coli* IMDH mutant relative growth rates] [34]	6	1	N.D.$^{\mathrm{c}}$	0.7566	< 0.00001***
Avian lysozyme thermostability [35]	3	1	4	0.5	<0.00001***
*N* = 5, *K* = 0	5	1	N.D.$^{\mathrm{d}}$	0.3333	<0.00001***
Log[relative fitness among *Methylobacterium extorquens* mutants] [10]	4	1	N.D.$^{\mathrm{c}}$	0.7527	0.00001***
Log[HIV replicative capacity on CCR5+ cells] [14]	5	3	25	0.5703	0.00002***
Log[cefotaxime MIC of *E. coli* TEM alleles] [66]	5	1	29	0.5490	0.00011**
Log[relative viability among fruit fly mutants] [70]	5	3	N.D.$^{\mathrm{c}}$	0.4896	0.00027**
Log[cefalexin MIC of *Bacillus cereus *metallo-$\upbeta$-lactamase alleles] [38]	4	1	N.D.$^{\mathrm{c}}$	0.5376	0.00448
*N* = 5, *K* = 1	5	2	N.D.$^{\mathrm{d}}$	0.5714	0.00474
Log[relative fitness among LTEE *E. coli* mutants in DM25 + EGTA] [20]	5	2	30	0.3486	0.01023
*N* = 5, *K* = 2	5	2	N.D.$^{\mathrm{d}}$	0.4428	0.0156
Log[relative colony growth rate among *Aspergillus niger* mutants] [16]	5	4	10	0.4387	0. 02002
Percent production of 5-epi-aristolochene by sesquiterpene synthase mutants [43]	6	10	N.D.$^{\mathrm{c}}$	0.1974	0.02639
Log[pyrimethamine IC50 of *P. falciparum* DHFR alleles assayed in *E. coli*] [33]	4	2	14	0.4337	0.03133
Log[IC75 of *E. coli* DHFR alleles against trimethoprim] [45]	6	2	55	0.1921	0.03639
*N* = 5, *K* = 4	5	5	N.D.$^{\mathrm{d}}$	0.1735	0.1442
Mammalian glucocorticoid receptor cortisol sensitivity [7]	4	4	N.D.$^{\mathrm{c}}$	0.1075	0.30182
Log[MIC of *E. coli* TEM alleles against ampicillin] [39]	4	3	N.D.$^{\mathrm{c}}$	0.0430	0.41356
*N* = 5, *K* = 5	5	7	N.D.$^{\mathrm{d}}$	−0.1632	0.86161

$^{\mathrm{a}}$Maximum possible value is 2$^{L}$

$^{\mathrm{b}}$Uncorrected value from permutation test (*n* = 10$^{5}$ replicates). Bonferroni-corrected *P* values: *** $\le$ 0.001; 0.001 < ** $\le$ 0.01; 0.01 < * $\le$ 0.05

$^{\mathrm{c}}$No data because no experimental variance estimates provided with this dataset

$^{\mathrm{d}}$No data because simulated fitness landscapes have no experimental variance

For each dataset, we then used a permutation test to test the null hypothesis that the corresponding correlation coefficient is zero. Specifically, each dataset is characterized by some number of epistatic terms: $2^{L}$ in cases where no experimental variance estimate is provided, or $2^{L}$ – *j* in cases where we were able to identify non-significant epistatic components (see previous paragraph and Table 1). For each of $n = 10^{5}$ replicates, we computed the rank correlation coefficient between two random permutations of this number (2$^{L}$ or $2^{L}$ – *j*) of the epistatic order values drawn from Eq. (2) for given *L*. We then sorted correlation coefficients, and the uncorrected *P* value reported for each dataset (Table 1) was taken as the fraction of permutations in which a correlation coefficient greater than or equal to the empirical value was observed. Thus, ours is a one-tailed test of the hypothesis that no positive correlation is present. This process is illustrated in Fig. 1c.

We used the Bonferroni–Holm method [24] to correct for multiple tests. In addition, under the null hypothesis that epistatic orders are uncorrelated with the naïve expectation given by Eq. (2), the distribution of *P* values observed across datasets should be uniformly distributed. We tested this hypothesis with a *G*-test after binning counts of empirically observed *P*values. We assessed statistical significance relative to the $\chi ^{2}$ distribution [56].

### Empirical Datasets

To compute all $2^{L}$ epistatic terms in a fitness landscape defined over *L* biallelic loci requires data on the fitness values (or suitable proxy) for each of the corresponding 2$^{L}$ genotypes. We previously designated such datasets combinatorially complete [67], and the datasets analyzed here are shown in Table 1. Several datasets [4, 34, 43, 45] had a few loci with cardinality greater than two. In these cases, we examined one “slice” through the landscape defined by randomly choosing just two alleles at those loci.

Several studies examined multiple phenotypes for a single set of mutations, and follow-up studies sometimes presented additional phenotypes for a previously described set of mutations. Those cases are enumerated in Table 2; for each set of mutations we randomly sampled just one phenotype. Table 2 also lists all combinatorially complete datasets we know that are defined over loci with cardinality greater than two. These were excluded here because the Fourier–Walsh framework doesn’t trivially generalize to higher cardinalities.

**Table 2:** Published combinatorially complete fitness landscapes not examined here

Phenotype [citation]	Number of loci	Number of genotypes$^{\mathrm{a}}$
Cycloguanil IC50 of *S. cerevisiciae* carrying *P. falciparum *DHFR alleles$^{\mathrm{b}}$ [11]	3	2$^{3}$ = 8
Pyrimethamine IC50 of *S. cerevisiciae* carrying *P. falciparum *DHFR alleles$^{\mathrm{b}}$ [8]	3	2$^{3}$ = 8
MIC against pyrimethamine of *S. cerevisiae* carrying *P. falciparum* DHFR alleles$^{\mathrm{b}}$ [8]	3	2$^{3}$ = 8
Pyrimethamine IC50 of *P. vivax* DHFR alleles assayed in *S. cerevisiae*$^{\mathrm{b}}$ [26]	4	2$^{4}$ = 16
MIC of TEM $\upbeta$-lactamase mutants against 15 $\upbeta$-lactams$^{\mathrm{b}}$ [39]	4	2$^{4}$ = 16$^\mathrm{{c}}$
*E. coli* operator binding affinities for 400 repressor sequences [48]	4	4$^{4}$ = 256
Relative fitness among LTEE *E. coli* mutants in DM25$^{\mathrm{b}}$ [29]	5	2$^{5}$ = 32
Relative fitness among LTEE *E. coli* mutants in DM25 + guanazole$^{\mathrm{b}}$ [20]	5	2$^{5}$ = 32
HIV replicative capacity on CXCR5+ cells$^{\mathrm{b}}$ [14]	5	2$^{5}$ = 32
Transcription factor/response element specificity in an ancient steroid hormone receptor [1]	5	4$^{2 }\times$ 2$^{3}$ = 128$^{\mathrm{d}}$
MIC of TEM-$\upbeta$-lactamase mutants against Piperacillin supplemented with clavulanic acid$^{\mathrm{b}}$ [63]	6	2$^{5}$ = 32
Diploid *S. cerevisiae* mutant growth rate$^{\mathrm{b}}$ [21]	6	2$^{6}$ = 64
Percent production of minor products by sesquiterpene synthase mutants$^{\mathrm{b}}$ [43]	6	2$^{6}$ = 64
Percent production of 4-EE by sesquiterpene synthase mutants$^{\mathrm{b}}$ [43]	6	2$^{6}$ = 64
Percent production of PSD by sesquiterpene synthase mutants$^{\mathrm{b}}$ [43]	6	2$^{6}$ = 64
104 mouse DNA-binding proteins’ affinity for 10 bp binding motifs [3]	10	4$^{10}$ = 1,048,576$^{\mathrm{e}}$
GFP affinity for 10 nucleotide base pair binding motifs [52]	10	4$^{10}$ = 1,048,576
Affinity of 1032 DNA-binding proteins spanning eukaryotic diversity against 10 nucleotide base pair binding motifs [69]	10	4$^{10}$ = 1,048,576$^{\mathrm{f}}$

$^{\mathrm{a}}$Written as the product of cardinalities across loci

$^{\mathrm{b}}$Another phenotype from this system is included in Table 1

$^{\mathrm{c}}$In total, 16 $\times$ 15 distinct $\upbeta$-lactam compounds = 240 observations are reported in this study

$^{\mathrm{d}}$This study examined all combinations of 4 nucleotides at two key positions in the DNA response element together with all combinations of two amino acids at three key positions in the transcription factor

$^{\mathrm{e}}$In total 1,048,576 $\times$ 104 DNA-binding proteins = 109,051,904 observations are reported in this study

$^{\mathrm{f}}$In total 1,048,576 $\times$ 1,032 DNA-binding proteins = 1,082,130,432 observations are reported in this study

Following [67], datasets reporting growth rates [4, 10, 14, 16, 20, 21, 70] and drug-resistance phenotypes [8, 33, 38, 39, 45, 66] were log-transformed before analysis. Following [45], negative two was used in place of log-transformed values when growth rate or drug resistance phenotypes of zero were observed. (In all cases, this is roughly one log order smaller than the smallest non-zero log-transformed value.) In cases where only mean and experimental variances (but not individual replicate observations) were provided, log transformations were approximated by Taylor expansions: ${\overline{{\hbox {ln}}(x)}}\approx \hbox {ln}({\bar{x}})-{s_x^2 }/{2 {\bar{x}}^{2}}$ and $s_{\mathrm{ln}(x)}^2 \approx \left( {{s_x }/{\bar{x}}} \right) ^{2}$. In cases where only means (but not variances) were provided, log transformations were approximated as ${\overline{{\hbox {ln}}(x)}}\approx \hbox {ln}( {\bar{x}} )$.

Following [45], for studies in which experimental variance estimates were provided, we recorded this quantity as a fraction of the total model variance. In one case [8], standard error was reported as standard error over “at least” two replicates; we therefore assumed *n* = 2 for each observation in that dataset. In one case [29], 95% experimental confidence intervals were reported, so variance estimates were computed under the assumption of normally distributed noise as $s^{2} = (n\cdot CI95/1.96)^{2}$.

### Simulated Fitness Landscapes

We used *NK* fitness landscapes [27] to test our hypothesis in a framework with explicitly tunable mutational interactions. Genomes in the *NK* model carry *N* loci. The fitness contribution of each locus depends on its allelic state and that at *K* others. Thus 0 $\le K \le N$ – 1 represents a parameter that tunes the level of epistatic interaction in the landscape. (See [41] and references therein for a number of elegant statistical properties of *NK* fitness landscapes.) We set *N*= 5 and generated one *NK *landscape for each 0 $\le K \le N$ – 1, where interacting loci were assigned at random in the genome. Simulated data were then analyzed as described in Fig. 1.

### Data and Software Archiving

Input data files, together with purpose-built MatLab code to perform all analyses described are archived at https://github.com/weinreichlab/JStatPhys2018. Kendall’s $\tau _{\mathrm{b}}$ correlation coefficient was computed using MatLab code developed elsewhere [9]. *NK *fitness landscapes were generated using code downloaded from https://github.com/qzcwx/NK-generator.

**Fig. 2:**
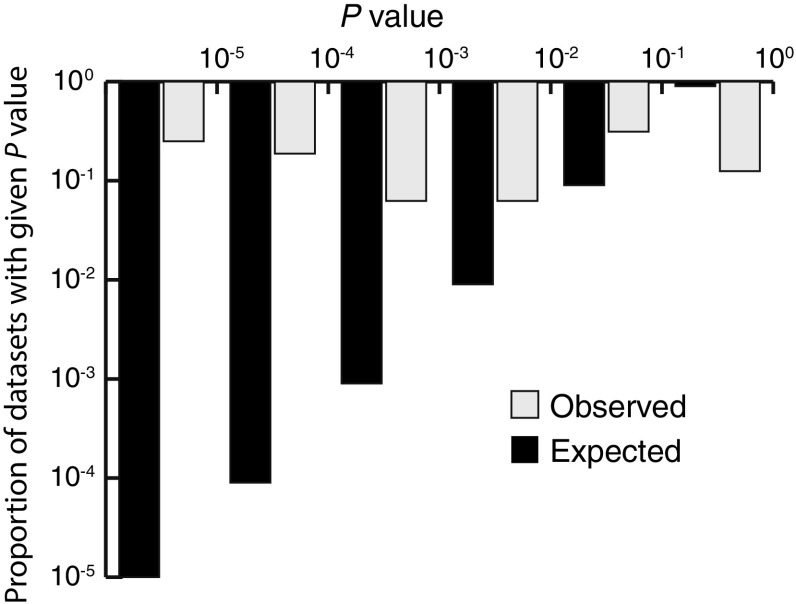
Distribution of uncorrected ${{\varvec{P}}}$ values among 16 empirical datasets. Under any null model, *P* values are expected to be uniformly distributed (black bars; note both axes are log-transformed). Instead observed *P* values (grey bars) are sharply skewed toward small values ($G = 143.77$, $P_{\mathrm{d.f.=5}} \ll 0.01$)

## Results

Epistasis can have profound consequences at many levels of biological organization [47, 53, 60, 71]. Here we tested the hypothesis that the influence of epistasis on empirical fitness landscape topography should decline as a function of epistatic order.

This study was originally stimulated by Fig. 2 in Palmer et al. [45], which examined six mutations in the dihydrofolate reductase (DHFR) gene of *E. coli *that contribute to increased resistance to an antimicrobial called trimethoprim. In that analysis, particular second- and third-order interactions were the third- and second-most influential epistatic terms for fitness landscape topography respectively. Indeed, just two of the first ten most influential epistatic terms were first-order, and in aggregate first-order terms explained just $\sim$ 28% of the variance in fitness across the landscape. At first blush, these results seem to challenge the hypothesis outlined in the previous paragraph, and we therefore sought to explore the pattern more broadly using published data from other systems.

Figure 1 illustrates the application of our analytic pipeline (see Sect. 2) to these same data. Our Fig. 1b closely recapitulates Fig. 2a in Palmer et al. [45]. While the precise sequence of epistatic terms differs slightly (likely because the previous study employed a subtly different framework for computing epistatic terms), higher-order epistatic interactions are again responsible for some the largest reductions in residual variance. Indeed, as previously observed, just two of the first ten terms are first-order, and in aggregate and first-order terms again explain just $\sim$ 28% of the variance in the data (Table 3a, compare the first two columns with Fig. 2b in [45]). Importantly however, Fig. 1c illustrates that we find a significant, positive correlation between expectation (Eq. 2) and the observed influence of epistatic terms on landscape topography as a function of their order ($\tau _{\mathrm{b}}$ = 0.1921, *P* = 0.03639).

We next applied our pipeline to 15 other published, combinatorially complete datasets. Results are summarized in Table 1 and shown graphically in Fig. S1. Out of all 16 datasets examined, 14 exhibit a significantly positive correlation between observation and the expectation, and eight of these remain significant after Bonferroni correction for multiple tests. Moreover, across datasets Table 1 exhibits a bias toward small *P* values. Under the null hypothesis (no significant correlation with expectation), we would expect a uniform distribution of *P* values. Instead, the observed distribution is sharply and significantly skewed toward small values (Fig. 2, *G* = 143.77, $P_{\mathrm{d.f.=5}} \ll$ 0.01).

We also applied our pipeline to *NK* fitness landscapes generated for *N* = 5 and 0 $\le K \le N$ – 1. We set *N* = 5 because the average size of the empirical datasets is 4.875 loci. Those results are also included in Table 1 (though omitted from Fig. 2).

## Discussion

Using a novel analytic pipeline (Fig. 1), we have examined 16 published, combinatorially complete biological datasets. This analysis broadly confirms our intuition that the influence of epistatic terms on empirical fitness landscape topography should decline with order, i.e., with the number of interacting mutations. Consistent with this intuition, observed fit to expectation in our simulated (*NK*) fitness landscapes deteriorates as the amount of epistasis (*K*) goes up. In the limit of $K=N$ – 1, fitness values (and hence, our epistatic terms) are i.i.d., and consequently the correlation is $\sim$ 0.

While considerable heterogeneity in effect exists among our empirical datasets (Table 1), eight of the 16 exhibit a Bonferroni-corrected, significantly positive correlation with expectation (Eq. 2). Moreover, across all 16 empirical datasets, we find a sharp bias toward significant *P* values (Fig. 2). Nor is there any correlation between the size of the dataset and uncorrected *P* value (not shown), suggesting that low statistical power is unlikely to contribute to the overall picture.

The relative magnitudes of epistatic terms depend on the underlying fitness scale employed [30, 67]. Although we log-transformed growth rate and drug resistance data (see Sect. 2.6), we have otherwise overlooked this fact. Recently, approaches for systematically rescaling data to minimize higher-order epistatic effects have been introduced [54] (see also [41, 62]). Applications of such methods would certainly have quantitative consequences for results presented here. However, because these approaches (on average) reduce higher-order epistatic terms, we believe this omission renders our conclusions conservative.

We also acknowledge that we failed to honor experimental uncertainty in the magnitudes of epistatic effects observed, which would almost certainly weaken the signal reported in Table 1. While we regard a rigorous treatment of experimental noise to be outside the scope of the present study, we note that the results presented in Fig. 2 are robust to its influence. Nevertheless, this is a serious concern for future consideration: because epistasis represents the difference between mutational fitness effects on different genetic background, experimental variance in fitness assays must be summed when computing variance in epistatic terms. For example, variance in epistatic terms computed with Eq. (1) will be roughly 2$^{L}$ as large as variance in the individual, underlying fitness measurements. Recently, an alternative, ranks-based approach to assessing epistatic interactions between mutations has been proposed [13], which appears to be less sensitive to this effect.

**Table 3:** Average epistatic influence on fitness landscape topography as a function of epistatic order in select datasets

Epistatic order	Aggregate reduction in residual variance	Number of epistatic terms significantly different from zero	Mean reduction in residual variance per epistatic term$^{\mathrm{a}}$
(a)	Log[IC75 of *E. coli* DHFR alleles against trimethoprim] [45]		
First	0.279	5	**0.056**
Second	0.266	12	0.022
Third	0.233	18	0.013
Fourth	0.144	11	0.013
Fifth	0.0685	6	0.011
Sixth	0.0065	1	0.0065
(b)	Mammalian glucocorticoid receptor cortisol sensitivity [7]		
First	0.171	4	0.043
Second	.405	6	0.067
Third	0.420	4	**0.105**
Fourth	0.004	1	0.004
(c)	Log[MIC of *E. coli* TEM alleles against ampicillin] [39]		
First	0.353	4	0.088
Second	0.278	6	0.043
Third	0.279	4	0.070
Fourth	0.091	1	**0.091**
(d)	*N* = 5, *K* = 4		
First	0.027	5	0.005
Second	0.315	10	0.315
Third	0.402	10	0.402
Fourth	0.241	5	**0.412**
Fifth	0.015	1	0.015

$^{\mathrm{a}}$Largest value for each dataset shown in bold

### The Combinatorics of Higher-Order Epistasis

This work was originally stimulated by a previous study [45] that examined six mutations in the DHFR gene responsible for increased trimethoprim resistance in *E. coli*. At first blush, results summarized in Fig. 2 of that study called into question the hypothesis that higher-order epistasis should only modestly influence naturally occurring fitness landscapes. And the salient features of that figure were recapitulated by our treatment (Fig. 1b, Table 3a).

However, our statistical analysis of those data reveals a strong positive correlation between epistatic influence on fitness topography as a function of epistatic order, consistent with our hypothesis (Fig. 1c). Thus in this system, the substantial influence of a few high-order epistatic terms is nevertheless consistent with the idea that high-order epistatic terms should in general only modestly contribute to fitness topography.

The resolution to this puzzle resides in the combinatoric number of epistatic terms. As noted above, given *L* biallelic loci there are $$\left( {{\begin{array}{l} L \\ k \\ \end{array} }} \right) $$ epistatic coefficients of order *k*, and this quantity grows almost exponentially for $k \ll L$. Indeed, after normalizing the summed influence of all epistatic terms of order *k* by the number of such terms, we observe that the per-term effect declines almost monotonically with order in this dataset (Table 3a; see also [67]). More generally, in all but three of the datasets examined, the normalized explanatory power is largest for first-order epistatic terms. Intriguingly, those three exceptions (see Table 3b-d: mammalian glucocorticoid receptor cortisol sensitivity [7], log[MIC of *E. coli* TEM allele sensitivity to ampicillin] [39] and the *N* = 5, *K* = 4 simulated fitness landscape) correspond to the three datasets with the largest *P* values in Table 1.

The consideration of the combinatorics of the problem is closely related to the Fourier spectrum of a fitness landscape [41, 57], namely the sum of squared epistatic coefficients as a function of interaction order. (This connection derives formally from the Appendix, which implies that the squared magnitude of each epistatic coefficient is monotonic in its influence on landscape topography.) The Fourier spectrum is proportional to the binomial coefficient when each genotype’s fitness is identically and independently distributed. This follows from the fact that on such landscapes all epistatic coefficients are also i.i.d., together with the combinatorics outlined in the previous paragraph. But as already anticipated by results in Table 3, the Fourier spectrum for the DHFR datasets is sharply shifted toward lower-order terms (not shown), as has previously been reported for both sesquiterpene synthase and several others biological datasets [41].

Nevertheless, declining average epistatic effects notwithstanding we find many examples of specific epistatic terms with anomalously large explanatory effects in many of the datasets examined here (Fig. S1). We suggest that these may reflect important mechanistic interactions among those particular mutations in the underlying biology of the system, thus representing potentially fruitful entry points for the molecular biologist [17].

### Epistasis and the Efficiency of Natural Selection

Our observation that the influence of epistatic terms on naturally occurring fitness landscapes declines with epistatic order raises the question of how epistatic terms influence the efficiency of natural selection. We lack a complete theoretical understanding of this connection.

One well-developed result concerns the influence of epistasis on the selective accessibility of mutational trajectories to high fitness genotypes. First, sign epistasis means that the sign of the fitness effect of a mutation varies with genetic background [68], and it renders selectively inaccessible at least some mutational trajectories to high fitness (e.g., [66]). But connections between sign epistasis and epistatic order are only now being developed [13]. Second, a subsetting approach similar to ours (Sect. 2.3) was recently used to examine the influence of epistatic interactions selectively accessible mutational trajectories to high fitness genotypes [55] in six of the datasets described here. Those authors found that higher-order terms indeed substantially alter the identity of selectively favored mutational trajectories to high-fitness genotypes, as well as their probabilities of realization. Further and consistent with findings here, that study also noted that the absolute magnitude of epistatic terms had an even larger effect on realized mutational trajectories than did their interaction order.

Moreover, pairwise epistasis has long been understood to influence not just the selective accessibility of high fitness genotypes but also the pace at which natural selection both increases the frequency of beneficial mutations (e.g., [18]) and at which it purges deleterious mutations (e.g., [31]). This work is closely related to the role that genetic recombination can play in “unlocking” epistatically interacting mutations (e.g., [5, 40]). However, to our knowledge the relationship between these effects and higher-order epistasis remains entirely unexplored.

In addition, we have only quantitatively examined the sequence of epistatic orders sorted by explanatory power (Fig. 1c). Thus, a great deal of information present in these data (e.g., the slopes in Figs. 1b and S1) remains to be examined. And of course, the number and size of available combinatorially complete datasets continues to grow, motivating further work in this regard. It seems reasonable to suppose that the development and testing of more nuanced theoretical predictions may be possible using data of the sort examined here.

Finally, we note that the Fourier–Walsh framework employed here depends on the availability of combinatorially complete datasets. But the experimental demands of this approach grow exponentially with the number of mutations examined. This fact sharply limits the scalability of analytic pipelines like ours. Recently, theoretical progress has been made in the analysis of less-than-complete datasets [6, 12, 13], and older work has also explored this idea [23, 58]. Theory that allows inferences using sparse datasets is likely to be a key advance in our ability explore broad, evolutionarily fascinating questions such as those considered here.

### Electronic supplementary material

Below is the link to the electronic supplementary material.

Supplementary material 1 (pdf 1923 KB)

## Appendix: The Explanatory Power of Fourier–Walsh Coefficients is Monotonic in Their Absolute Magnitude

Assume two fitness functions defined over *L* biallelic loci are represented as column vectors $\overrightarrow{W}$ and $\vec {X}$ with Fourier–Walsh coefficients $\overrightarrow{E_{W}}$ and $\overrightarrow{E_{X}}$ computed with Eq. (1). Define the sum of squares distance between $\overrightarrow{W}$ and $\vec {X}$ as $\Vert \overrightarrow{W}-\vec {X} \Vert \equiv \sum _{i=1}^{{2}^{L}}(W_{i}-X_{i})^2=(\overrightarrow{W}-\vec {X})^{\mathrm{T}}\cdot \overrightarrow{W} -\vec {X}$, where $w_{\mathrm{i}}$ and $x_{\mathrm{i}}$ are the $i{\mathrm{th}}$ components of $\overrightarrow{W}$ and $\vec {X}$, respectively.

### Theorem 1

Sum of squares distance equivalence

$$\begin{aligned} \Vert \overrightarrow{W}-\vec {X}\Vert =2^{L} \Vert \overrightarrow{E_{W}}-\overrightarrow{E_{X}} \Vert . \end{aligned}$$

### Proof

By definition

$$\begin{aligned} \overrightarrow{W}= {\varvec{\Psi }} \overrightarrow{E_{W}} \hbox { and } \vec {X} = {\varvec{\Psi }} \overrightarrow{E_{X}} \end{aligned}$$

where ${\varvec{\Psi }}$ is the Hadamard matrix (see Sect. 2.2).

Therefore

$$\begin{aligned} \Vert \overrightarrow{W}-\vec {X}\Vert= & {} \Vert {\varvec{\Psi }}\overrightarrow{E_{W}} - {\varvec{\Psi }} \overrightarrow{E_{X}}\Vert \\= & {} \left( {\varvec{\Psi }}\overrightarrow{E_{W}}-{\varvec{\Psi }}\overrightarrow{E_{X}} \right) ^{\mathrm{T}}\cdot \left( {\varvec{\Psi }}\overrightarrow{E_{W}}-{\varvec{\Psi }} \overrightarrow{E_{X}}\right) \\= & {} \left( \left( {\varvec{\Psi }}\overrightarrow{E_{W}}\right) ^{\mathrm{T}}-\left( {\varvec{\Psi }}\overrightarrow{E_{X}}\right) ^{\mathrm{T}}\right) \cdot \left( {\varvec{\Psi }} \overrightarrow{E_{W}}-{\varvec{\Psi }}\overrightarrow{E_{X}} \right) \\= & {} \left( \left( \overrightarrow{E_{W}}\right) ^{\mathrm{T}}{\varvec{\Psi }}^{\mathrm{T}}-\left( {\overrightarrow{E_{X}}} \right) ^{\mathrm{T}}{\varvec{\Psi }}^{\mathrm{T}} \right) \cdot \left( {\varvec{\Psi }}\overrightarrow{E_{W}}-{\varvec{\Psi }}\overrightarrow{E_{X}}\right) \\= & {} \left( \left( \overrightarrow{E_{W}} \right) ^{\mathrm{T}}-\left( \overrightarrow{E_{X}} \right) ^{\mathrm{T}} \right) {\varvec{\Psi }} ^{\mathrm{T}}\cdot {\varvec{\Psi }}\left( \overrightarrow{E_{W}}-\overrightarrow{E_{X}}\right) \end{aligned}$$

But recall that ${\varvec{\Psi }}^{\mathbf{T}}{{\varvec{\Psi }} }=2^{L}{} \mathbf{I},$ where **I** is the identity matrix. Thus

$$\begin{aligned} \Vert \overrightarrow{W} -\vec {X}\Vert= & {} 2^{L}\left( \left( \overrightarrow{E_{W}}\right) ^\mathrm{T} -\left( \overrightarrow{E_{X}}\right) ^\mathrm{T}\right) \cdot \left( \overrightarrow{E_{W}}- \overrightarrow{E_{X}}\right) \\= & {} 2^{L}\left( \overrightarrow{E_{W}}-\overrightarrow{E_{X}} \right) ^{\mathrm{T}}\cdot \left( \overrightarrow{E_{W}}-\overrightarrow{E_{X}} \right) =2^{L}\Vert \overrightarrow{E_{W}}-\overrightarrow{E_{X}}\Vert . \end{aligned}$$

$$\square $$

An interesting property of the Hadamard matrix is that ${\varvec{\Psi }} ^{\mathrm{T}}=2^{L}{\varvec{\Psi }}^{-1}$. Without the 2$^{L}$ this equality is the hallmark of a rotational transformation. This means that Fourier–Walsh coefficients are simply the result of a high dimensional axis rotation of the coordinates of function space, together with a uniform contraction. This provides intuition into Theorem 1: rotating the space and contracting it uniformly only changes the distance between two vectors in the space by the constant of contraction.

### Theorem 2

Minimizing the sum-of-squares distance of subsetting approximations

The subsetting approximation $\overrightarrow{W^{(m)}}={{\varvec{\Psi }} }\overrightarrow{E_{W}^{(m)}}$ that minimizes the sum of squares distance to function $\overrightarrow{W}$ is the one whose $\overrightarrow{E_W^{(m)}}$ uses the *m*   largest components in absolute value in $\overrightarrow{E_{W}}=\frac{1}{2^{L}}{\varvec{\Psi }} \overrightarrow{W}$.

### Proof

By Theorem 1, the sum of squares distance between $\overrightarrow{W}$ and $\overrightarrow{W^{\left( m \right) }}$ is $\Vert \overrightarrow{W}-\overrightarrow{W(m)}\Vert = 2^{L}\Vert \overrightarrow{E_{W}} - \overrightarrow{E_{W}^{(m)}}\Vert$, which means that we can equivalently solve the minimization problem on either side of the equality. And trivially, the right-hand side is minimized when the *m* nonzero components in $\overrightarrow{E_{W}^{\left( m \right) } }$ are the *m* largest components in absolute value in $\overrightarrow{E_{W}}$. (The squaring of differences in epistatic terms in the definition of $\Vert \overrightarrow{E_{W}}-\overrightarrow{E_{w}^{\left( m \right) } }\Vert$ removes the significance of their sign.) $\square$
