# Supplementary figures and images for: The Influence of Higher-Order Epistasis on Biological Fitness Landscape Topography
Source: J Stat Phys. 2018 Feb 7;172(1):208–25. doi: 10.1007/s10955-018-1975-3 (PMC5986866; doi:10.1007/s10955-018-1975-3)

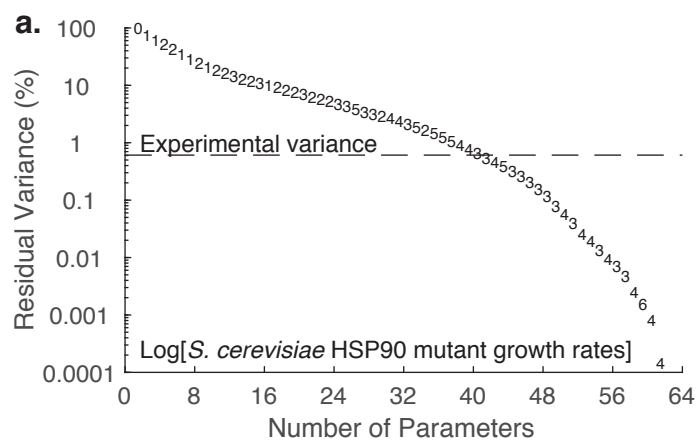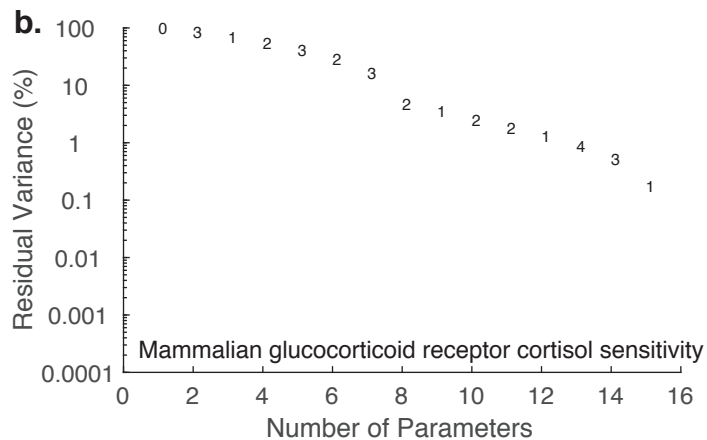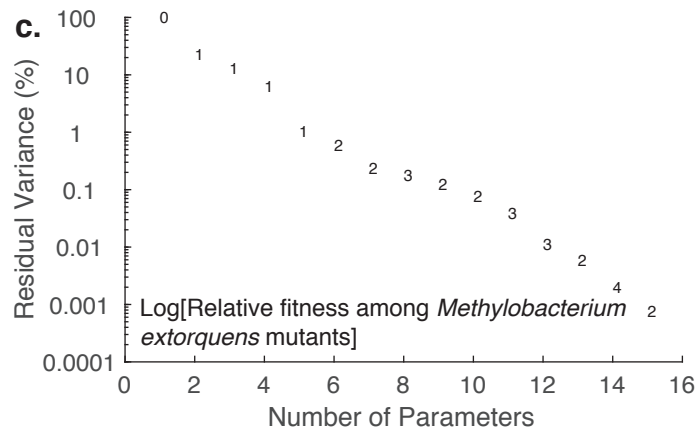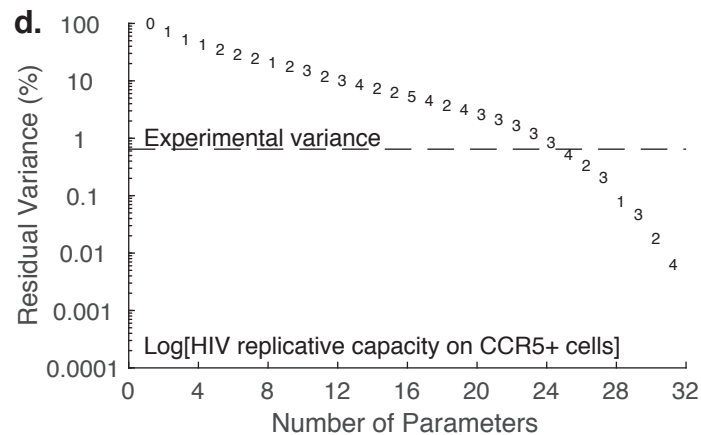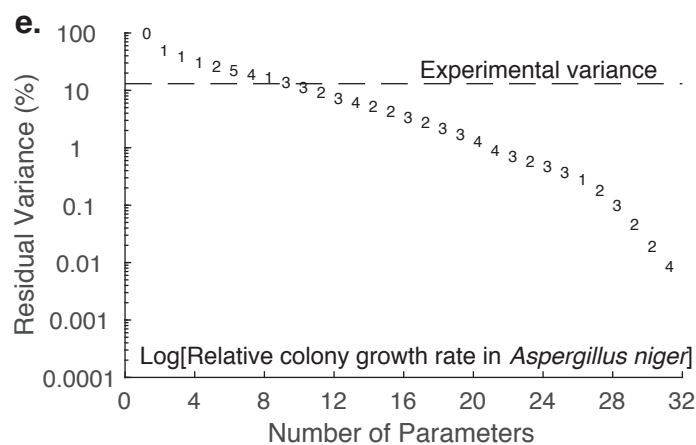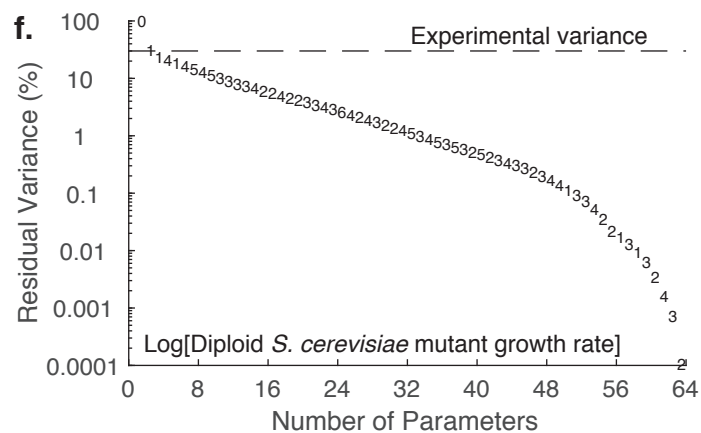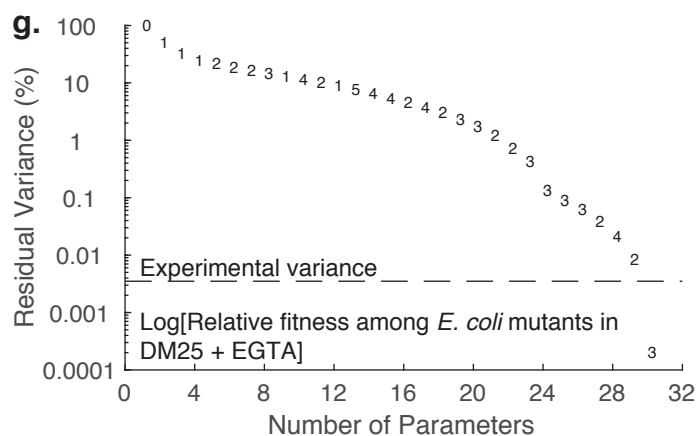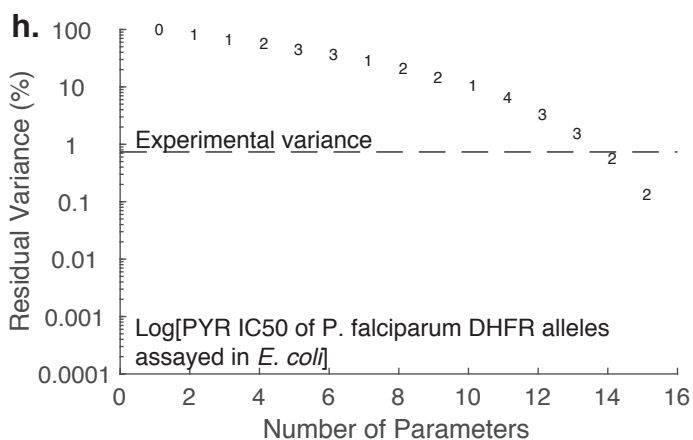

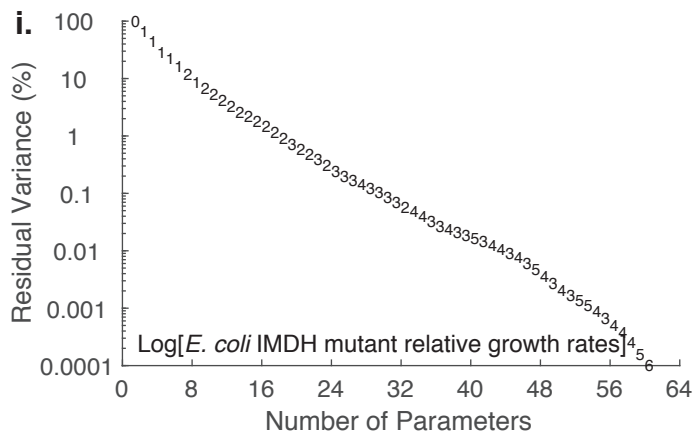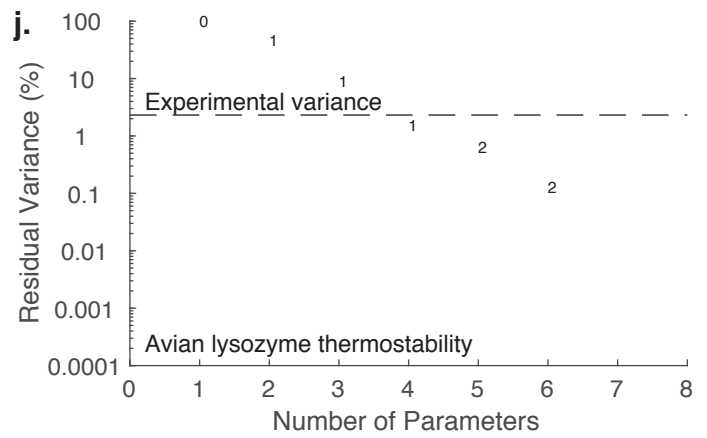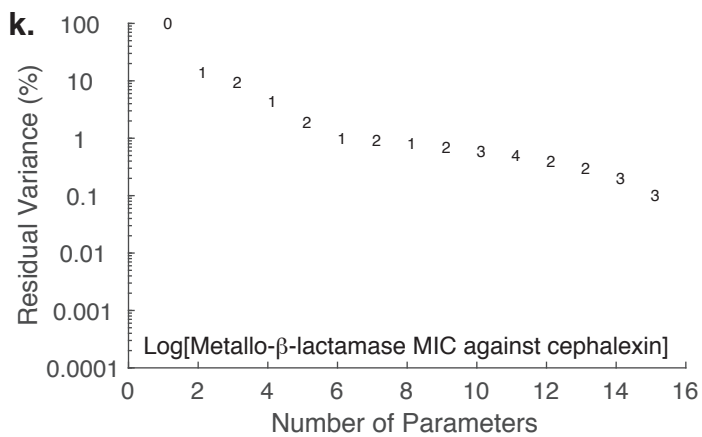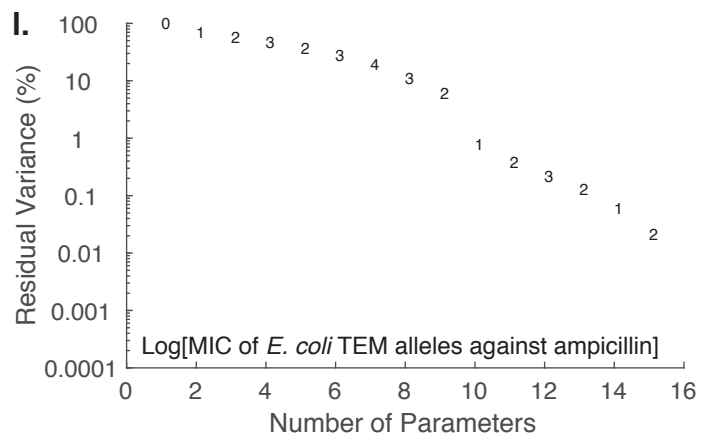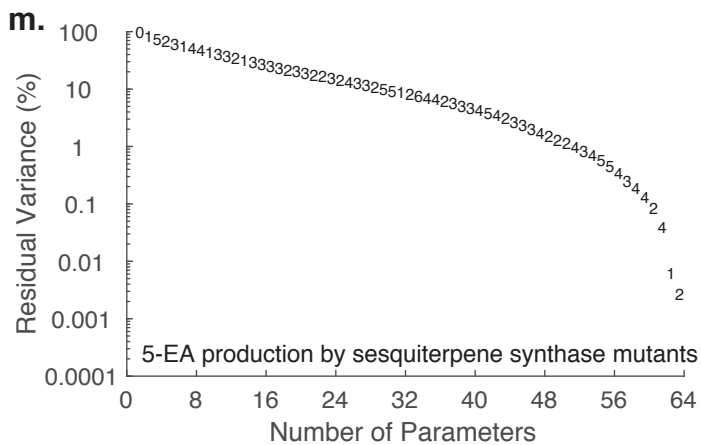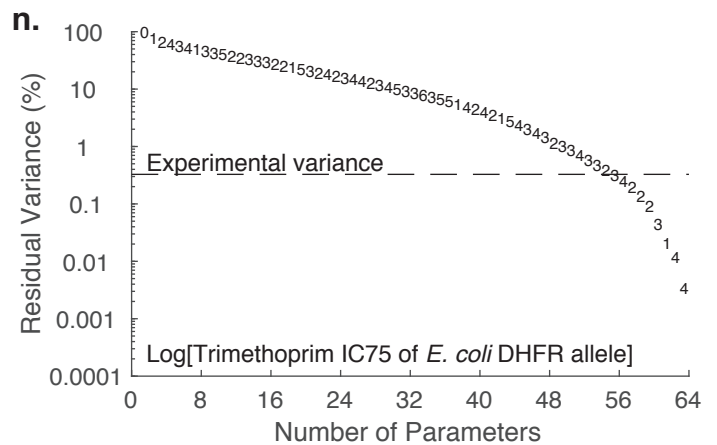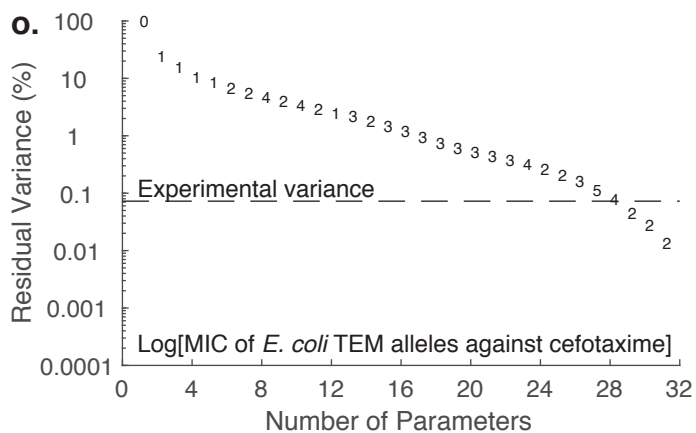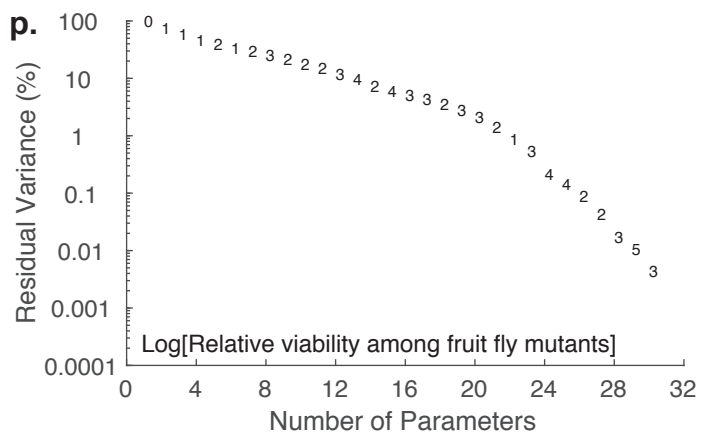

Supplement: Supplementary file 1 — Supplementary material 1 (pdf 1923 KB) [file 10955_2018_1975_MOESM1_ESM.pdf]
